# Acupuncture for cognitive functions in post-COVID-19 condition: study protocol of a three-armed, randomized controlled trial with multimodal MRI

**DOI:** 10.3389/fmed.2026.1796351

**Published:** 2026-04-29

**Authors:** Tingting Luo, Yang Luo, Dafeng Liu, Hongjiao Jin, Yi An, Jinwei Huang, Kun Luo, Yan Guo, Dan Wang, Liang Huang, Xi Wu

**Affiliations:** 1 Acupuncture and Tuina College, Chengdu University of Traditional Chinese Medicine, Chengdu, China; 2 Department of Rehabilitation, The Thirteenth People’s Hospital of Chongqing, Chongqing, China; 3 Department of Pediatrics, Chongqing Health Center for Women and Children, Women and Children’s Hospital of Chongqing Medical University, Chongqing, China; 4 Department of Science and Teaching, Public Health Clinical Center of Chengdu, Chengdu, China

**Keywords:** acupuncture, brain network, cognitive function, magnetic resonance imaging, multimodal approach, neuropsychological testing, post-COVID-19 condition, topology

## Abstract

**Background:**

Post-COVID-19 condition (PCC), commonly called long COVID, is a prevalent sequela of SARS-CoV-2 infection and can affect multiple organ systems. Cognitive dysfunction is one of the most common symptoms in PCC, with a prevalence of 22%. It can persist for years and significantly reduce patients’ quality of life. The brain network is the neural basis underlying human cognitive processes. Diffusion tensor imaging (DTI) and functional magnetic resonance imaging (fMRI) studies have revealed that cognitive impairments across attention, memory, executive function, and language are associated with alterations in network characteristics for PCC. Currently, there is no accepted therapy for cognitive impairment in PCC. Acupuncture has the potential to improve cognitive deficits in PCC. This trial aims to investigate the effect of acupuncture on cognitive functions in patients with PCC, and to explore the underlying mechanism of its effects on cognition in this condition using DTI and fMRI.

**Methods:**

In this three-armed, randomized controlled trial, 117 PCC patients with cognitive symptoms will be randomly assigned in a 1:1:1 ratio to verum acupuncture (VA), sham acupuncture (SA), or a waitlist control group. Participants in the VA and SA groups will receive three sessions of treatment per week for 8 weeks. The primary outcome measures are the changes in Addenbrooke’s Cognitive Examination-III (ACE-III) total score and phonemic fluency test score at week 8. The secondary outcome measures include the Digit Span Test (DST), Symbol Digit Modality Test (SDMT), Trail Making Test (TMT), Rey’s Auditory Verbal Learning Test (RAVLT), Rey-Osterrieth Complex Figure Test (RCFT), Stroop Color Word Test (SCWT), category fluency test, action fluency test, and Boston Naming Test (BNT-30), as well as global and regional topological measures of structural and functional brain networks constructed from DTI and fMRI data. Additionally, the Fatigue Severity Scale (FSS), the Generalized Anxiety Disorder-7 (GAD-7), the 24-item Hamilton Depression Scale (HAMD-24), and the MOS 36-item Short Form Health Survey (SF-36) will also be measured.

**Discussion:**

The results of this study will reveal the effect of acupuncture treatment on cognitive functions for PCC and provide insights into the mechanisms by which acupuncture may improve cognition in PCC.

**Clinical trial registration:**

ClinicalTrials.gov (www.clinicaltrials.gov), identifier: NCT07355751.

## 1 Introduction

Post-COVID-19 condition (PCC), commonly called long COVID, refers to the symptoms that occur during probable or confirmed SARS-CoV-2 infection, or develop after initial recovery, persist at least 3 months after COVID-19 onset, and last for at least 2 months, and cannot be explained by an alternative diagnosis (1). According to the estimates of the World Health Organization (WHO), 10–15% COVID-19 survivors are affected by PCC worldwide (2). PCC consists of diverse manifestations, among which cognitive dysfunction is one of the most common symptoms, affecting approximately 22% of the post-COVID-19 population (3). Cognitive problems can last for years; furthermore, the symptom burden appears to increase over time from 6 months to 2–3 years after infection onset, manifesting as a worsening of pre-existing cognitive symptoms and the emergence of new-onset symptoms (4). In addition to self-reported cognitive dysfunction, objective deficits in multiple cognitive domains were also observed among patients with PCC, including attention, memory, executive function, language, and visuospatial ability (5–9). The subjective and objective cognitive impairments have a significant impact on patients’ quality of life (10, 11).

Brain networks are the neural basis that support cognitive processes and complex behaviors in humans (12, 13) and are shaped by both the physical white matter tracts connecting regions (structural connectivity) and the patterns of communication between them (functional connectivity) (14). Diffusion tensor imaging (DTI) and functional magnetic resonance imaging (fMRI) are important tools for investigating structural and functional brain networks, respectively. The former visualizes the white matter (WM) tracts that connect two brain regions by measuring the direction and magnitude of water molecule diffusion in the brain and quantifying WM microstructural changes. Additionally, an anatomical network can be constructed at the whole-brain level based on diffusion imaging and fiber tract imaging techniques (15). fMRI measures brain activity through detecting alterations in blood flow and oxygenation, and functional connectivity (FC) can be estimated by analyzing temporal correlations of neural activity between two regions. Similarly, a functional network of the entire brain can be constructed by calculating the functional connections between each pair of brain regions (16). Multiple studies have revealed microstructural alterations across broad WM areas among patients with PCC, including decreased fractional anisotropy (FA) and axial diffusivity (AD), and increased mean diffusivity (MD) and radial diffusivity (RD) (7, 17–19). Moreover, these changes were related to the impaired performances in verbal fluency, concentration, and information processing speed (7, 20, 21). Several resting-state fMRI (rs-fMRI) studies for PCC reported disrupted FCs between hippocampus and parietal areas; between prefrontal regions and cerebellum and occipital lobes; between bilateral parahippocampus (22, 23); and enhanced FCs between striatum and parietal cortex; between cerebellum and resting-state networks (RSNs) (24, 25); furthermore, altered FCs in cerebellum and hippocampus are associated with performances of memory, cognitive control, and working memory among individuals with PCC (20, 21). The graph theoretical analysis for functional network revealed alterations of regional topological measures (nodal centrality, betweenness centrality, and closeness centrality) in multiple nodes such as olfactory gyrus, orbitofrontal and cingulate cortices, and thalamus in patients with PCC relative to healthy people, suggesting regional reorganization of functional network, and there were significant links between these changes and performances in divided attention, verbal memory tests for PCC patients (26, 27).

The investigation into the treatment of cognitive dysfunction in PCC is still in its infancy. The efficacy of medication, cognitive rehabilitation, and non-invasive brain stimulation remains uncertain due to limited and mixed evidence (28–32). Acupuncture, as a Traditional Chinese Medicine (TCM) therapy, has played a positive role in treating cognitive dysfunction associated with multiple neurological and neuropsychiatric disorders, such as mild cognitive impairment (MCI), stroke, and insomnia (33–35). In addition, two case reports showed that acupuncture treatment significantly improved the cognitive symptoms of PCC patients (36, 37). Currently, there are several acupuncture studies on PCC being conducted (NCT06476496, NCT05890508, NCT06633666, NCT05212688, NCT06144320, ChiCTR2300076060, ChiCTR2300068708), only a few of which focus on cognitive outcome; moreover, a single cognitive indicator is evaluated for most studies.

Acupuncture has the potential to modulate brain networks. Wang et al. found that 14 sessions of acupuncture treatment significantly increased FA values in the right cerebral peduncle, anterior limb of the internal capsule, posterior corona radiata, and the cingulum-hippocampus for mild traumatic brain injury (mTBI) with postconcussion symptoms in comparison with the sham acupuncture group using DTI, and the increase of FA values was associated with improvement of postconcussion symptoms (38). Similarly, the positive modulatory effect of acupuncture treatment on WM integrity was also observed by Shen and colleagues for patients with cerebral infarction (39). For functional brain networks, it has been found that acupuncture at bilateral Zusanli (ST36) increased global network efficiency of the functional network constructed based on rs-fMRI data for healthy subjects (40). A systematic review (44 rs-fMRI studies) on modulatory effects of acupuncture on RSFCs across healthy people and patients with nervous system diseases, motor system diseases, and other diseases showed that acupuncture could increase default mode network (DMN), sensorimotor network (SMN) connectivity with pain-, affective-, and memory-related brain areas and resulted in greater connectivity between the periaqueductal gray (PAG), anterior cingulate cortex (ACC), left posterior cingulate cortex, insula, limbic, paralimbic, and precuneus compared with sham acupuncture (41). Additionally, Tan et al. revealed that increased RSFCs between cognition-related regions such as the insula, dorsolateral prefrontal cortex, hippocampus, thalamus, inferior parietal lobule, and anterior cingulate cortex following acupuncture were positively related to improvement of cognitive performances among patients with MCI (42). However, it remains unclear whether acupuncture treatment can improve cognitive function through modulating brain networks. At present, an rs-fMRI study focusing on the effect of acupuncture on FCs within the dorsal attention network (DAN) and DMN for PCC patients with MCI is being conducted in China (ChiCTR2400092961). However, there is no such study that investigates the underlying mechanisms of acupuncture improving cognitive function for PCC at the whole-brain network level.

In this context, this randomized controlled trial with DTI and rs-fMRI aims to evaluate the effect of acupuncture treatment on cognitive functions for patients with PCC. The second objective is to determine whether acupuncture improves cognitive functions for PCC through modulating the organization of brain networks.

## 2 Methods and design

### 2.1 Study design

**Figure 1:**
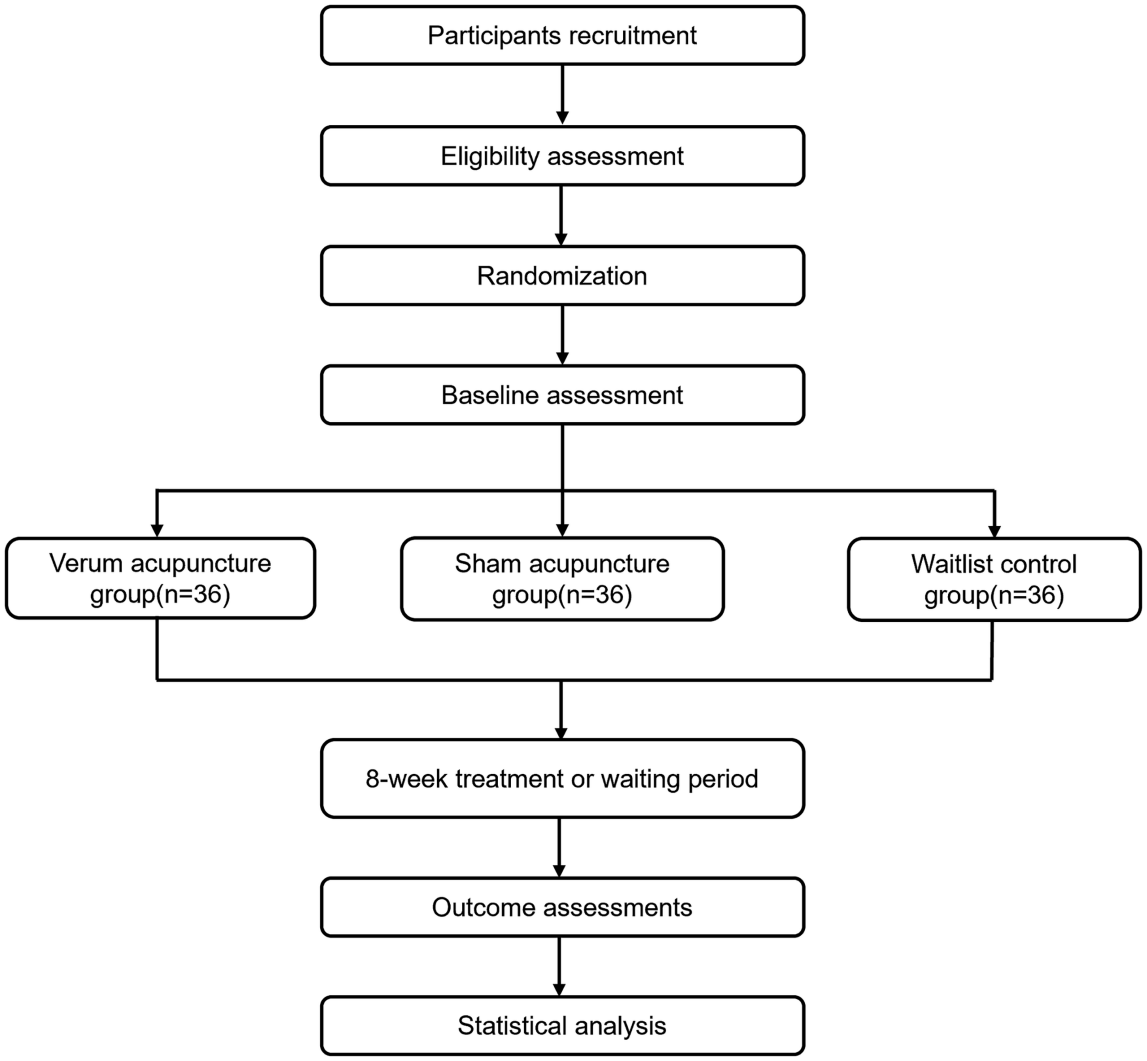
Flowchart of study procedure.

This study is a three-armed, randomized controlled trial with DTI and rs-fMRI. A total of 117 PCC adults with cognitive symptoms will be randomly assigned to verum acupuncture, sham acupuncture, or a waitlist control group in a 1:1:1 ratio. This trial will be cooperatively completed by two hospitals in Chengdu. Participants for this trial will be prospectively recruited from three sources: the COVID-19 survivors who were hospitalized in the Public Health Clinical Center of Chengdu from 01 January 2020 to 31 December 2023, the Affiliated Hospital of Chengdu University of Traditional Chinese Medicine, and the communities in Chengdu. For the hospitalized COVID-19 survivors, two researchers will initially screen potential participants through a phone interview. Then, a face-to-face interview and routine blood tests will be performed to screen eligible participants. For the latter two sources, recruitment advertisements will be disseminated both online and offline using platforms such as WeChat, websites, and others. The interventions and assessment will be carried out in the outpatient department of the Affiliated Hospital of Chengdu University of Traditional Chinese Medicine. Patients and the public will not be involved in the design, conducting, and report of this trial. Figure 1 illustrates the study flowchart.

### 2.2 Eligible criteria for participants

#### 2.2.1 Diagnostic criteria

The diagnosis of PCC is made according to the criteria developed by WHO (1): (1) a history of probable or confirmed SARS-CoV-2 infection, (2) symptoms occur during the initial illness or after initial recovery from an acute COVID-19 episode lasting for at least 2 months at 3 months from the infection onset, and (3) the symptoms cannot be explained by an alternative diagnosis.

#### 2.2.2 Inclusion criteria

People who meet all of the following criteria will be included: (1) age 18–60 years; (2) fulfilling WHO criteria for PCC; (3) history of confirmed COVID-19 through RT-PCR of nasopharyngeal swab or antigen test of nasal swab; (4) having subjective cognitive problems; (5) native Chinese speakers; (6) right handedness; (7) voluntary to participate in the study and sign the informed consent form.

#### 2.2.3 Exclusion criteria

Those who meet any of following criteria will be excluded: (1) preexisting cognitive symptoms before COVID-19; (2) previous or current diagnosis of chronic conditions that may affect cognitive performances, such as neurological, psychiatric disorders, organ failure, and chronic infectious diseases; (3) prior loss of consciousness; (4) history of major surgery within a year; (5) long-term use of drugs that may influence cognition, such as tranquilizer, antidepressant, and immunosuppressor; (6) previous acupuncture treatment within 3 months; (7) involvement of other clinical study within 4 weeks; (8) obesity(BMI ≥ 28); (9) sensory disorders(deafness, color blindness); (10) limb dysfunction; (11) metal implantation; (12) claustrophobia; (13) pregnancy or lactation.

### 2.3 Withdrawal criteria

Participants with any of the following conditions will be withdrawn: (1) poor compliance, unwillingness to continue in the study, or voluntary withdrawal; (2) severe adverse events or complications, making continuation unsuitable; (3) failing to adhere to the treatment protocol or having insufficient observation data, which influenced the assessment.

### 2.4 Interventions

All interventions will be carried out in the outpatient department of the Affiliated Hospital of Chengdu University of Traditional Chinese Medicine. A trained and certified acupuncturist with at least 5 years of experience will deliver interventions. The treatment consists of 24 sessions of 30 min, given within 8 weeks (three sessions per week). The acupuncturist will twirl and lift or thrust needles for 10 s every 15 min during each treatment session. The acupuncturist is not allowed to discuss the acupuncture effect with patients during the treatment period.

#### 2.4.1 Verum acupuncture group

Based on the trial version of Traditional Chinese Medicine Rehabilitation Guidance and Suggestions for convalescent COVID-19 survivors issued by the National Health Commission of the People’s Republic of China and the National Administration of Traditional Chinese Medicine, and the analysis of acupoints selection in the acupuncture treatment for MCI (42). This study selected two sets of acupoints for the verum acupuncture group. The first set of acupuncture points includes Baihui (GV20), Shenting (GV24), bilateral Neiguan (PC6), Qihai (CV6), Guanyuan (CV4), bilateral Zusanli (ST36), and bilateral Sanyinjiao (SP6). The second set of acupoints consists of Sishenchong (EX-HN1), bilateral Ganshu (BL18), bilateral Pishu (BL20), and bilateral Shenshu (BL23). A set of acupoints will be acupunctured in each treatment session, and the two sets of acupoints are alternatively selected. The locations of acupoints conform to the 2021 National Standards of the People’s Republic of China (GB/T12346-2021) and are presented in Table 1.

**Table 1:** Locations and needling methods for acupoints used in the verum acupuncture group.

Acupoints	Locations	Needling methods
Baihui (GV20)	On the top of the head, 5 cun above the middle point of the anterior hairline.	Subcutaneous insertion 0.5–0.8 cun twirling needle to elicit Deqi sensation.
Shenting (GV24)	On the front part of the head, 0.5 cun above the middle point of the anterior hairline.	Subcutaneous insertion 0.5–0.8 cun, twirling needle to elicit Deqi sensation.
Neiguan (PC6)	On the medial area of the forearm, 2 cun above the distal wrist crease, between the palmaris tendon and the radial wrist flexor tendon.	Perpendicular insertion 0.5–1 cun, twirling or lifting and thrusting needles to elicit Deqi sensation.
Qihai (CV6)	On the lower abdominal area, 1.5 cun below the navel, at the anterior midline.	Perpendicular insertion 1–1.5 cun, twirling or lifting and thrusting needles to elicit Deqi sensation.
Guanyuan (CV4)	On the lower abdominal area, 3 cun below the navel, at the anterior midline.	Perpendicular insertion 1–1.5 cun, twirling or lifting and thrusting needles to elicit Deqi sensation.
Zusanli (ST36)	On the outer side of the lower leg, 3 cun below the outer knee point, and a middle finger breadth to the anterior border of the tibia.	Perpendicular insertion 1–2 cun, twirling or lifting and thrusting needles to elicit Deqi sensation.
Sanyinjiao (SP6)	On the medial side of the shank, 3 cun above the tip of the medial malleolus, by the posterior medial edge of the tibia.	Perpendicular insertion 1–1.5 cun, twirling or lifting and thrusting needles to elicit Deqi sensation.
Sishenchong (EX-HN1)	On the top of the head, 1 cun lateral to Baihui (GV20) at anterior, posterior, left, and right directions, a total of 4 points.	Subcutaneous insertion 0.5–0.8 cun, twirling needles to elicit Deqi sensation.
Ganshu (BL18)	On the back, below the spinous process of the 9th thoracic vertebra, 1.5 cun away from the posterior midline.	Oblique insertion 0.5–0.8 cun toward the spine, twirling needles to elicit Deqi sensation.
Pishu (BL20)	On the back, below the spinous process of the 11th thoracic vertebra, 1.5 cun away from the posterior midline.	Oblique insertion 0.5–0.8 cun toward the spine, twirling needles to elicit Deqi sensation.
Shenshu (BL23)	On the lower back, below the spinous process of the 2nd lumbar vertebra, 1.5 cun away from the posterior midline.	Perpendicular insertion 0.5–1 cun, twirling or lifting and thrusting needles to elicit Deqi sensation.

Participants will lie in a supine or prone position while receiving verum acupuncture treatment. The disposable sterile acupuncture needles (0.30 × 40 mm, Hwato brand, Suzhou Medical Supplies Factory Co., Ltd., Suzhou, China)are used for treatment. The needling method for each acupoint is summarized in Table 1.

#### 2.4.2 Sham acupuncture group

**Figure 2:**
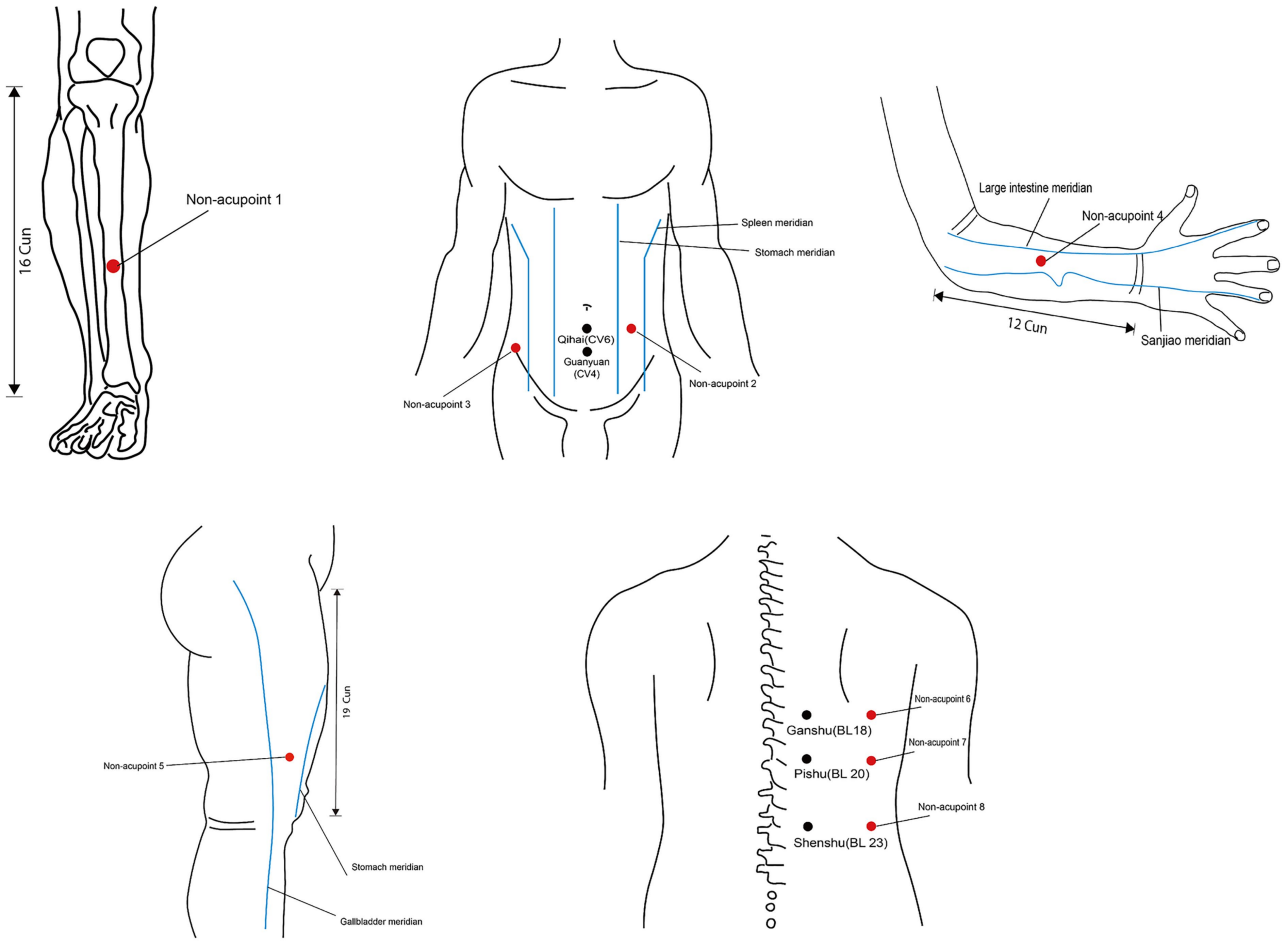
Locations of non-acupoints.

**Figure 3:**
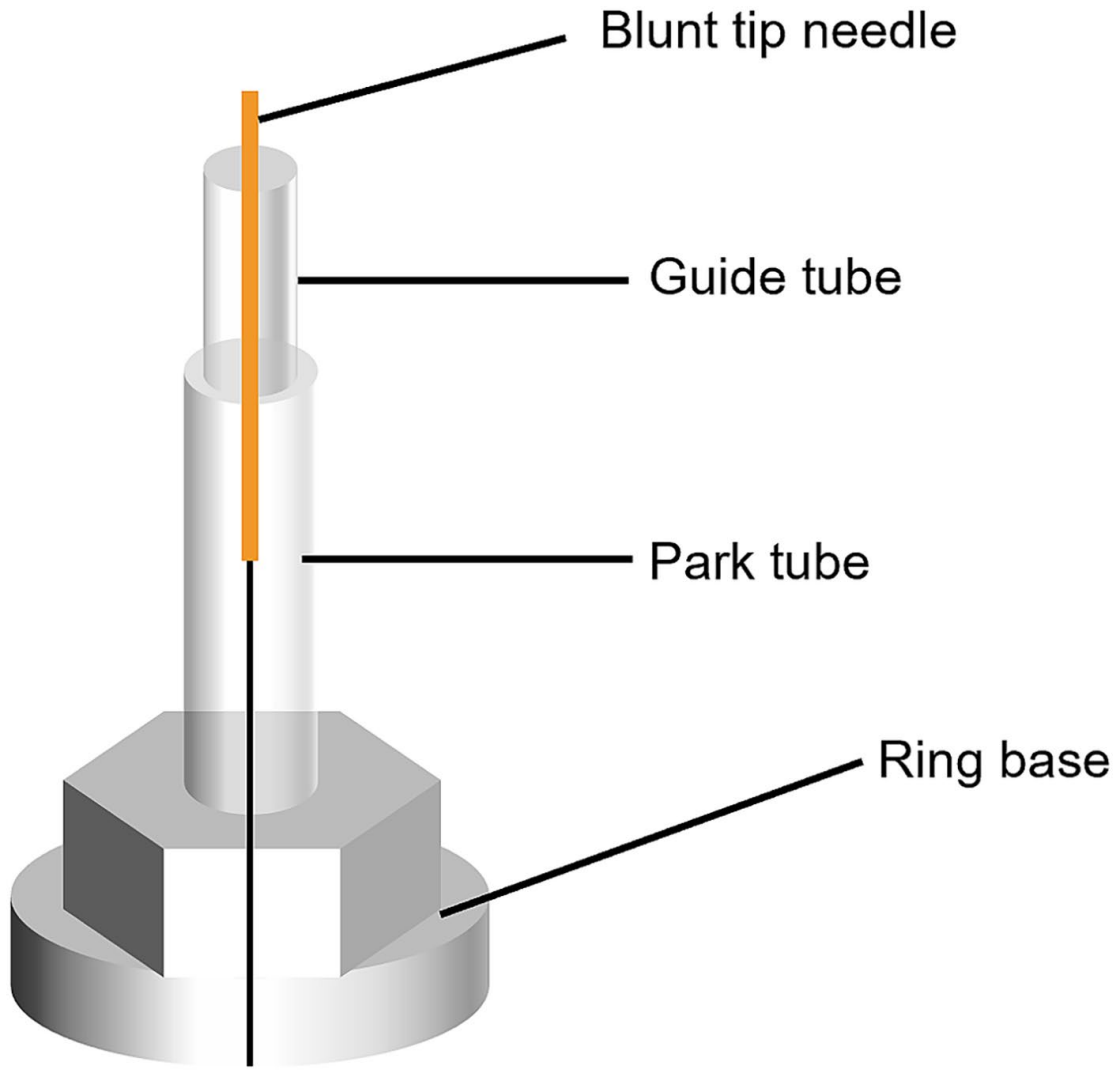
Park sham acupuncture device.

Corresponding to the verum acupuncture group, nonpenetrating acupuncture on non-acupoints will be performed using a Park sham acupuncture device (0.25 mm in diameter and 40 mm in length, Hwatuo, Suzhou, China) for participants in the sham acupuncture group. The same as the acupuncture group, two sets of non-acupoints will be alternatively acupunctured. The first set of sham acupoints includes bilateral non-acupoint 1, non-acupoint 2, non-acupoint 3, bilateral non-acupoint 4, and bilateral non-acupoint 5. The second set consists of bilateral non-acupoint 6, bilateral non-acupoint 7, and bilateral non-acupoint 8. The location of non-acupoints is presented in Table 2 and Figure 2. Figure 3 illustrates the Park sham acupuncture device.

**Table 2:** Locations of non-acupoints used in the sham acupuncture group.

Non-acupoints	Locations
Non-acupoint 1	In the central area of the tibia, 6 cun above the tip of the lateral malleolus.
Non-acupoint 2	On the lower abdominal area, the left side of Qihai (CV6), the midpoint between the stomach meridian and spleen meridian.
Non-acupoint 3	On the lower abdominal area, 6 cun right lateral to Guanyuan(CV4).
Non-acupoint 4	On the outer side of the forearm, 6 cun above the wrist crease, and between the large intestine meridian and the Sanjiao meridian.
Non-acupoint 5	On the thigh area, 4 cun above the patellar base, the middle point between the stomach meridian and gallbladder meridian.
Non-acupoint 6	On the back, 3 cun lateral to Ganshu.
Non-acupoint 7	On the back, 3 cun lateral to Pishu.
Non-acupoint 8	On the back, 3 cun lateral to Shenshu.

During acupuncturing, participants will lie in a supine or prone position, and the Park sham device will be stuck on the skin of sham acupoints through the ring base. The blunt and retractable needle will be inserted into the plastic tube, and the needle will retract into the handle when the blunt tip contacts the skin. Thus, there will be no Deqi sensation.

#### 2.4.3 Waitlist control group

Participants in the waitlist control group are instructed not to receive any therapy during the 8-week waiting period, but a regular health follow-up through phone will be delivered once a week by an appointed investigator. For those presenting deterioration of symptoms during the waiting period, symptomatic intervention will be given. For those developing a new condition, they will be recommended to withdraw from the study for relevant treatment. Participants who complete an 8-week waiting period will receive the same acupuncture treatment as the verum acupuncture group for free.

### 2.5 Sample size calculation

G Power 3.1 software is used to calculate sample size. The changes in Addenbrooke’s Cognitive Examination-III (ACE-III) total score and phonemic fluency test score are the primary outcomes in this study. Based on previous clinical trials on cerebral small vessel disease cognitive impairment (CSVDCI) (43), idiopathic trigeminal neuralgia, and our pilot study findings, we estimate that the mean±SD of changes in ACE-III for verum acupuncture, sham acupuncture, and waitlist groups at 8 weeks after randomization are, respectively, 4 ± 1, 3.5 ± 2.7, and 0.5 ± 1.2, and the mean±SD of changes in phonemic fluency test are, respectively, 3.8 ± 2, 2.5 ± 1, and −1.1 ± 1, setting a one-sided significance level of 5% and power of 80% in a 1:1:1 ratio. In total, 14 cases per group are needed for ACE-III, and 34 patients per group are required for the phonemic fluency test. Estimating a 15% drop out rate, a total of 117 patients need to be recruited in this study.

### 2.6 Randomization

An appointed investigator who is not involved in participant recruitment, treatment, outcome assessment, and data analysis will generate a random sequence using SAS version 9.4 software (SAS Institute, Inc) through a simple random method. The acupuncturists who are not involved in participants’ recruitment will obtain the randomized allocation sequence and group from this investigator by short message service (SMS).

### 2.7 Blinding

The primary investigator, participant recruitment researchers, outcome assessors, data analysts, and participants in verum acupuncture and sham acupuncture groups will be blinded to treatment allocation. It is difficult to blind patients in the waitlist control group, because they do not receive treatment. Acupuncture and sham acupuncture treatments will be performed in separate treatment rooms to reduce the risk of accidental unblinding. A participant’s treatment will be revealed if severe adverse events (SAEs) occur during the trial.

To test the success of masking, participants in verum acupuncture and sham acupuncture groups will be asked to guess which group they were in at the end of treatment (week 8). The Bang’s blinding index (Bang’s BI) will be calculated based on the guessed proportion of each group (44, 45).

### 2.8 Neuropsychological assessment

**Figure 4:**
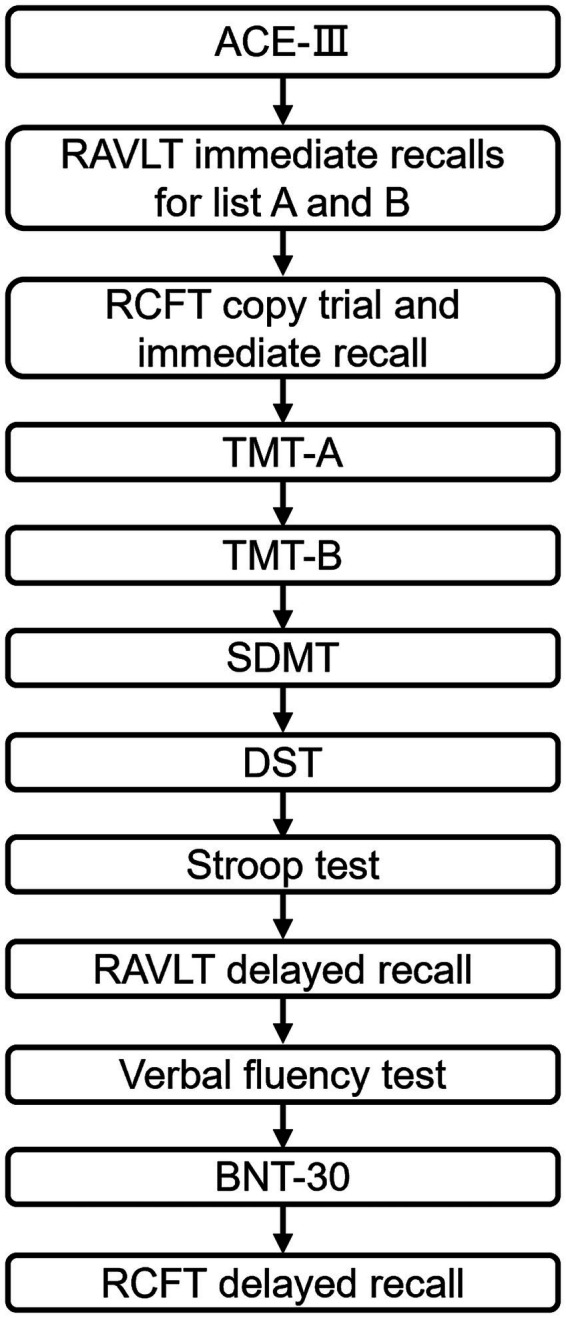
Administration order of cognitive tests.

Two trained investigators will perform the neuropsychological assessment in a quiet room with suitable light and temperature. Subjects are instructed not to take medicines that may affect cognition (such as tranquilizers and antidepressants), coffee, or alcohol 1 day before the assessment, and to have a good night’s sleep. The neuropsychological assessment will be performed 2 h after breakfast with validated Chinese version tests. Figure 4 shows the administration flowchart of the neuropsychological tests.

Addenbrooke’s Cognitive Examination-III(ACE-III) will be used to evaluate general cognitive function. Attention will be assessed with the Digit Span Forward Test (DST-F), Symbol Digit Modality Test (SDMT), and Trail Making Test Part A (TMT-A). Memory will be evaluated with Rey’s Auditory Verbal learning (RAVLT), immediate and delayed recall trials of the Rey-Osterrieth Complex Figure Test (RCFT). The Trail Making Test Part B(TMT-B), Digit Span Backward Test (DST-B), and Stroop Color Word Test (SCWT) will be used to assess executive functions. Language will be assessed with the phonemic fluency test, category fluency test, action fluency test, and 30-item Boston Naming Test (BNT-30). Copy trial of RCFT will be applied to evaluate visuospatial ability. Those neuropsychological tests are selected because impaired performances have been reported in patients with PCC, especially those with subjective cognitive problems, in comparison with healthy people without COVID-19 history and recovered COVID-19 survivors.

### 2.9 Imaging data acquisition

MRI scans will be performed at Huaxi MR Research Center of West China Hospital. An experienced radiologist will acquire brain images using a 3.0 T PRISMA scanner (Siemens, Erlangen, Germany) with a 64-channel head coil. All participants will undergo a T2-weighted fluid-attenuated inversion recovery (FLAIR) scan to exclude cerebral organic lesions (such as stroke and tumor) prior to formal experimental scans. After exclusion of cerebral organic lesions for each participant, the high-resolution T1-weighted imaging (T1w), DTI, and rs-fMRI data will be acquired in sequence. During imaging data acquisition, subjects are instructed to relax and stay awake with closed eyes, and foaming pads will be used to reduce head motion.

T1-weight images will be acquired using a 3D magnetization prepared rapid gradient echo (3D-MPRAGE) sequence with the following parameters: repetition time (TR):1500 ms, echo time (TE): 1.87 ms, flip angle = 10°, field of view (FOV): 256 mm × 256 mm, slice thickness: 0.8 mm, acquisition time: 7.15 min. DTI data will be obtained using a single-shot echo planar imaging (SS-EPI) sequence with the following parameters: 127 diffusion directions, TR: 4300 ms, TE: 87 ms, FOV:216 mm × 216 mm, slice thickness:1.8 mm, b = 0, 1,000, 2000, 3,000 s/mm^2^. The scan parameters for the rs-fMRI images are as follows: gradient-echo echo-planar imaging (GRE-EPI) sequence, TR: 2000 ms, TE: 37 ms, slice thickness: 2 mm, FOV: 208 mm × 208 mm.

### 2.10 Imaging data preprocessing

#### 2.10.1 DTI data

DTI images for each participant will be preprocessed using the PANDA toolbox^1^ based on FMRIB Software Library (FSL 5.0).^2^ The main steps include (1) converting the DTI images in DICOM to NIFTI format; (2) extracting the b0 image; (3) correcting eddy current and head motion by registering the DTI images to the b0 image; (4) stripping non-brain tissue; (5) fitting the diffusion tensor model at the voxel level using TMRIB diffusion toolbox (FDTV 3.0); and calculating the FA value for each voxel.

#### 2.10.2 Rs-fMRI data

The rs-fMRI data for all participants will be preprocessed using the Graph Theoretical Network Analysis (GRETNA) toolbox according to following steps: (1) removing the first 10 rs-fMRI images to allow for signal stabilization; (2) slice timing correction; (3) realignment for head motion correction (excluding participants with head motion > 2 mm or head rotation > 2°); (4) normalizing functional images into Montreal Neurological Institute (MNI) space through the DARTEL alignment method and resampled to a voxel size of 3 × 3 × 3 mm^3^; (5) regression of nuisance covariates (including Friston-24 head motion parameters, global mean signal, white matter signals, and cerebrospinal fluid signals); (6) bandpass filtering (0.01–0.08 Hz) for the time series of each voxel to remove low frequency drift and high frequency noise.

### 2.11 Brain networks construction

#### 2.11.1 Structural network

The structural network will be constructed with the PANDA toolbox. First, the entire brain image will be first segmented into 90 cortical and subcortical regions using the Automated Anatomical Labeling (AAL) template, and each segmented region is regarded as a node of the whole-brain structural network. Next, the FA image in native space is co-registered to a T1-weighted structural image using an affine transformation for each participant. Then, the transformed T1-weighted images are nonlinearly registered to the Montreal Neurological Institute space (MNI-152). After that, the inverse transformation parameters are used on the AAL atlas to generate the corresponding regions in individual spaces. Subsequently, fiber tracking and whole-brain fiber construction are performed using the fiber assignment based on the continuous tracking (FACT), and tracking stops while FA < 0.2 or the turning angle >45°. If the number of WM fibers is ≥2, then the two regions are considered structurally connected. Finally, a 90 × 90 network matrix for each participant will be constructed, within which the network edge is defined as the mean FA value of WM fibers connecting two regions.

#### 2.11.2 Functional network

The GRETNA toolbox will be applied to construct a whole-brain functional network for each patient. The AAL atlas is used to divide the brain into 90 regions, with each region representing a network node. The Pearson correlation coefficients of the mean time series between all pairs of the nodes are calculated, representing the edges of the network. Then, a 90 × 90 functional connectivity matrix is constructed for subsequent network analysis for each participant. The correlation matrix is transformed into an undirected binary matrix with the threshold values ranging from 0.1 to 0.34 with intervals of 0.01.

### 2.12 Outcomes

#### 2.12.1 Primary outcome measures

The primary outcome measures are the changes of ACE-III total score and phonemic fluency test score at week 8.

#### 2.12.2 Secondary outcome measures

(1) Cognitive measures: DST-F, TMT-A, SDMT, RAVLT, RCFT, TMT-B, DST-B, SCWT, category fluency test, action fluency test, and BNT-30.

(2) Imaging measures: The graph theoretical analysis will be performed to quantify the topological properties of structural and functional networks using the GRETNA toolbox. The global and regional measures will be assessed: (1) clustering coefficient (Cp): the average of clustering coefficients across all nodes in a network, reflecting the density and complexity of the entire network; (2) characteristic path length (Lp): the average of shortest path length (the minimum number of edges required to connect one node to another node) across any pair of nodes in the network, measuring the speed of information transmission in the network; (3) global efficiency(Eg): the mean of inverse of the shortest path length between all pairs of nodes in a network, quantifying the functional integration of the network; (4) local efficiency (Eloc): the mean of the local efficiency across all nodes in whole network, indicating the stability of a local network when the local network is interrupted; (5) normalized clustering coefficient (*γ*): the ratio of Cp to Cprand (the Cp of a random network); (6) normalized characteristic path length (*λ*): the ratio of Lp to Lprand (the Lp of a random network); (7) small-worldness (*σ*): the ratio of γ to λ, reflecting the balance between local specification and global integration of information transmission; (8) degree centrality (Ndc): the number of direct connections attached to a node, measuring the information transmission between a node and other network nodes; (9) nodal efficiency (Ne): the average of the inverse of shortest path length between a given node and all other nodes, representing the efficiency of information transmission between a node and other nodes; (10) betweenness centrality (Nbc): the number of times a given node lies on one of the paths between all pairs of nodes in a network, reflecting the influence of a node on the overall flow of information in the network; (11) closeness centrality (Ncc): the reciprocal of the sum of the shortest path lengths from that node to all other nodes, quantifying how close a node is to all other nodes in a network; (12) nodal clustering coefficient (Ncp): the number of a node’s neighbors (nodes directly connecting the node) that are also connected, reflecting the extent of “cliquishness” of the subnetwork where the node is located; (13) nodal local efficiency (Nle): inverse of shortest path length between all node pairs in the sub-network formed by a given node and its neighbors, measuring the efficiency of information transmission between the node and its neighbors; (14) nodal shortest path length (Nlp): the average of the shortest path length from a single node to all other nodes in a network, quantifying the efficiency of information transmission of the node. Among these metrics, clustering coefficient(Cp), characteristic path length (Lp), local efficiency (Eloc), global efficiency (Eg), normalized clustering coefficient(*γ*), normalized characteristic path length (*λ*), and small-worldness (*σ*) are global measures. Small-world property is evaluated by γ, λ, and σ. Degree centrality (Ndc), nodal efficiency (Ne), betweenness centrality (Nbc), closeness centrality (Ncc), nodal clustering coefficient (Ncp), nodal local efficiency (Nle), and nodal shortest path length (Nlp) are local topological parameters.

(3) Mental health and quality of life: According to the consensus regarding core outcome set (COS) and COS measurement instruments for PCC in adults released by WHO (43, 44). The Fatigue Severity Scale (FSS), the Generalized Anxiety Disorder-7 (GAD-7), the 24-item Hamilton Depression Scale (HAMD-24), and the MOS 36-Item Short Form Health Survey (SF-36) will also be measured at baseline and week 8.

### 2.13 Adverse events and safety

Any Adverse Events (AEs) or SAEs that occur during the intervention, whether related to the intervention or not, will be recorded in case report forms (CRFs) in detail (including the onset pattern, onset time, duration, severity, frequency, and treatment). Investigators will judge the relationship between AEs or SAEs and acupuncture treatment according to information including cause, time, and course. Common AEs related to acupuncture (including fainting, stuck needles, broken needles, bent needles, bleeding, edema, and other minor AEs) will be treated by the acupuncturist immediately. For SAEs, such as stroke recurrence or organ damage, researchers will immediately stop the treatment and send the patient to the emergency department. SAEs will be reported to the ethics committee within 24 h. The research group will bear treatment costs for AEs and SAEs.

### 2.14 Data collection and management

Two trained investigators will collect the demographic data, medical history, blood test results, and clinical assessment data through CRFs. The data of each participant will be entered into a database, which is created based on CRF by EpiData software (version 3.1) and managed by an appointed investigator. The neuroimaging data will be stored in a portable hard drive.

### 2.15 Statistical methods

An appointed statistician who is blind to the grouping of participants will perform the statistical analysis using R software (version 4.2.1; R Foundation). Normal continuous variables will be described as mean±standard deviation, while non-normally distributed data will be described as median and quartile [M(P25 P75)]. The categorical variable will be described as a percentage. *p*-value<0.05(one-tailed) is set as the significance level for all analyses.

All analyses will follow the Intention-to-Treat (ITT) protocol. The ITT analysis includes all participants who are randomly assigned to the groups, regardless of their adherence to the entry criteria, the treatment they actually received, or deviation from the treatment protocol. The missing data will be handled by multiple imputations. Per-protocol (PP) population analysis will also be performed for participants who have finished the whole treatment protocol after randomization.

First, comparisons for demographic data (such as age, gender, and educational level), illness duration, estimated premorbid intelligence function, and Bang’s BI will be conducted to examine the comparability across the three groups. The categorical data will be analyzed using the chi-square test. Normally distributed continuous variables will be tested using one-way analysis of variance, whereas the non-normal data will be analyzed using the Kruskal–Wallis test.

The primary outcomes will be compared by an analysis of covariance (ANCOVA). The ANCOVA will be adjusted by age, education, estimated premorbid intelligence ability, and Bang’s BI. In addition, a mixed-effects model for repeated measures will be performed to verify the findings. For secondary outcomes, a mixed-effects model for repeated measures will be performed. An ANCOVA with the adjustment of age, education, estimated premorbid intelligence ability, and Bang’s BI at the end of treatment will be used as a secondary analysis to assess the robustness of the findings. Additionally, participants will be divided into those with MCI (ACE-III ≤ 85) and those without MCI (ACE-III > 85) according to their ACE-III total score (46), and a subgroup analysis within each stratum will be performed to examine whether there is a differential acupuncture effect for patients across different cognitive status groups. The multiple comparisons for primary and secondary outcomes will be corrected with the Benjamini–Hochberg procedure with a significance setting at *p* < 0.05. Chi-square test will be used to compare the rate of AEs and SAEs between groups. Finally, Spearman correlation analysis will be performed to examine the relationships between those cognitive outcome measures and imaging indicators exhibiting inter-group differences.

## 3 Discussion

To the best of our knowledge, this is the first randomized controlled trial that aims to systematically investigate the efficacy of acupuncture treatment on cognitive functions in PCC and its central mechanism using multimodal MRI techniques.

PCC is a multisystemic disease with diverse symptoms. Cognitive dysfunction is one of the most common debilitating symptoms for PCC (11). Neuropsychological evidence revealed that PCC was associated with deficits in general cognitive function and five core cognitive domains defined by the Fifth Edition of the Diagnostic and Statistical Manual of Mental Disorders (DSM-V), including attention, memory, language, executive function, and visuospatial ability (5, 6, 18, 24, 47).

Brain networks are the neural basis underlying complex cognitive processes, consisting of numerous regions and edges (WM tracts and FCs) that connect them together; the attributes of brain networks can be investigated at the whole-brain level using multimodal neuroimaging techniques, such as DTI and fMRI (48). PCC patients presented significant connectivity alterations in wide brain regions, including WM microstructures (7, 17–19, 21, 49, 50), inter-regional FCs (21–24), and topological properties of functional network (21, 22, 26, 51), and these changes were associated with performances in multiple cognitive domains (21, 22, 26, 51).

Currently, accepted therapy for cognitive dysfunction in PCC is lacking (52). Acupuncture, as a TCM therapy, has exerted a positive role in improving cognitive impairments for multiple neurological and neuropsychiatric disorders (34, 35, 53, 54). Additionally, network neuroscience evidence suggested that acupuncture could increase cognitive performance through modulating the organization of brain networks such as MCI, Alzheimer’s disease (AD), and stroke (34, 42, 55). However, it is still unclear whether acupuncture treatment can increase cognitive performance through modulating brain networks properties for PCC.

In this study, two sets of acupoints will be alternatively acupunctured to overcome acupuncture tolerance (repeated or prolonged stimulation of an acupoint leads to the attenuation or disappearance of the therapeutic effect of acupuncture treatment) (56, 57), which is a common phenomenon in clinical practice, and then maintain the sensitivity and responsivity of patients to acupuncture treatment. Although the alternating protocol may introduce variability in the acupuncture effect, this protocol is fixed within the group, so that patients in the acupuncture group will receive identical acupuncture treatments. This variability, caused by the alternating needling of different acupoints, is systematic and is considered a part of the acupuncture effect. There is no trial that uses an alternating protocol to examine the effect of acupuncture on cognitive function, but cognitive enhancement has been identified in other disorders for each acupoint set used in this study (58, 59). The findings of this study are able to provide evidence on the feasibility of alternating acupuncture in clinical practice and acupuncture trials.

This study intends to include sham acupuncture and waitlist controls to comprehensively reveal the therapeutic effect of acupuncture. The comparison between verum and sham acupuncture could determine the specific therapeutic effect of acupuncture, and the contrast to the waitlist group can assess the overall effect of acupuncture. The non-penetrating acupuncture on non-acupoints will be used as a sham control in this trial, and this method could completely exclude the factors that play vital roles in the specific therapeutic effect of acupuncture, including acupoint specificity and needling sensation (60, 61), but adequately control for the non-specific effect of acupuncture. Nevertheless, we should acknowledge that the difference in needling sensation between non-penetrating acupuncture and penetrating acupuncture may result in the failure of blinding, especially for individuals having acupuncture experience (62). Therefore, the success of blinding will be tested in verum acupuncture and sham acupuncture groups, and the blinding assessment will be included in statistical analysis to eliminate the influence of the difference on outcomes.

Objective neuropsychological tests are selected as outcomes in this study. These tests are chosen because impaired performances have been detected in patients with PCC (5–8, 18, 63, 64) and have a wide application in clinical practice (65). Particularly, a deficit in phonemic fluency has been consistently reported by all neuropsychological studies on PCC (6–8, 18, 63, 64); thus, this test is applied as a primary outcome in this study.

Regarding brain networks, graph-based analysis will be used to systematically examine the global and regional topological attributes of structural and functional networks. This is helpful to identify neural targets of acupuncture treatment for PCC. Moreover, this study can gain insight into the neural mechanism of cognitive changes following acupuncture treatment through correlation analysis between cognitive and brain network responses to acupuncture treatment.

This study also explores the effect of acupuncture treatment on fatigue, anxiety, depression, and quality of life using FSS, GAD-7, HAMD, and SF-36. These outcomes are selected according to the core outcome set recommendation for adults, PCC developed by WHO (66). These results may provide some directions for future research.

There are several limitations to this study. First, the study sample size is relatively small, which may reduce the ability to detect statistical intergroup differences in outcomes, especially in imaging metrics, for which larger samples are required to survive multiple-comparison correction. Even so, this trial is valuable as a basis for future large-scale studies. Second, as a result of an unestablished cognitive signature and the lack of objective diagnostic criteria for cognitive dysfunction in PCC, the definition of cognitive impairment in PCC patients relies solely on subjective reports rather than objective cognitive assessment in this study, which may result in a ceiling effect. To address this issue, subgroup analyses of patients with and without cognitive impairment, classified according to the ACE-III total score, will be included in this study. Third, rigorous eligibility criteria are applied in this study, with the exclusion of any conditions that may influence cognitive performance, which may limit the generalizability of the study results. Fourth, because of the nature of acupuncture, blinding the acupuncturists and participants in the waiting-list control group is impossible. Therefore, participant recruitment researchers, outcome assessors, and data analysts will be masked from group assignments in this study. Finally, this is a monocentric study that recruits participants from Chengdu. The patients included in this study may not represent patients in other regions of China or among foreigners living in Western countries.

Taken together, we anticipate that acupuncture treatment may improve cognitive performance for patients with PCC through modulating the speed and efficiency of information transmission and functional integration within brain structural and functional networks. The results of this study will not only reveal the effect of acupuncture treatment on objective cognitive function in patients with PCC but also provide insights into the underlying neural mechanism of acupuncture in improving cognitive function in this disease, which ultimately may provide a new treatment option for PCC patients with cognitive dysfunction.

## Footnotes

1
https://www.nitrc.org/projects/panda

2
https://www.nitrc.org/projects/fsl
